# Supplementary material for: Melvin is a conversational voice interface for cancer genomics data
Source: Commun Biol. 2024 Jan 5;7:30. doi: 10.1038/s42003-023-05688-z (PMC10770357; doi:10.1038/s42003-023-05688-z)
Supplement: Supplementary file 2 — Supplementary Information PDF [file 42003_2023_5688_MOESM2_ESM.pdf]

Supplementary Information

Supplementary Notes

Supplementary Note 1: Accessing Melvin and Additional Resources
- Melvin Skill
- Melvin Terms of Use and Privacy Policy
- Melvin Documentation
- Custom Pronunciation Web Portal

Supplementary Note 2: Brief anatomy of an Alexa Skill
- Conversation Initiation
- Automatic Speech Recognition (ASR)
- Natural Language Understanding (NLU)
- Interaction Model
- Text-to-speech
- Speech Synthesis Markup Language (SSML)
- Voice Recording and Privacy

Supplementary Note 3: Interaction Model
- Interactions
- Supported Data Types

Supplementary Note 4: Intent Handler
Supplementary Note 5: Navigation State Tracking
Supplementary Note 6: Out-of-vocabulary Mapper Service (OOVMS)
- Pronunciation Quiz Skill
- Custom Pronunciation Web Portal

Supplementary Note 7: Data Explorer Service
- Database design

Supplementary Note 8: End-to-end Testing Framework
Supplementary Note 9: Enhancing User Experience

Supplementary Tables

1. Supported intents within Melvin
2. Supported data types within Melvin
3. Supported interactions within Melvin
4. Examples of possible compare and split-by analyses

Supplementary Figures

1. Cloud-based system design of Melvin
2. Crowdsourcing utterances through the Pronunciation Quiz Alexa skill
3. Utterance processing workflow for the Out-of-vocabulary Mapper Service (OOVMS)
4. Creating user-specific utterance mappings through Custom Pronunciation Web Portal
5. Melvin deployed in a laboratory meeting or molecular tumor board setting

Supplementary Notes

Supplementary Note 1. Accessing Melvin and Supporting Resources

Here we provide access to the following:

Melvin Skill: The Melvin skill can be accessed via the following URL or QR code: https://www.amazon.com/dp/B09NZSRBNS

Melvin Terms of Use and Privacy Policy: Melvin's Terms of Use can be found here: https://pittgenomics.gitlab.io/melvin_docs/docs/policies/terms/

Melvin's Privacy Policy can be found here: https://pittgenomics.gitlab.io/melvin_docs/docs/policies/privacy/

Melvin documentation: Melvin's documentation – including supported intents, animated demonstration videos, and FAQ – can be found here: https://www.melvin.pittlabgenomics.com/docs/

Custom Pronunciation Web Portal: The Custom Pronunciation Web Portal can be accessed here: https://www.melvin.pittlabgenomics.com/record

Supplementary Note 2. Anatomy of an Amazon Alexa Skill

Here we briefly describe native components of the Amazon Alexa platform that are integral for understanding Melvin's design, engineering, and usage.

Conversation Initiation: There are two approaches to initiating a conversation with Alexa, which is determined by the type of device where it runs. On smartphones, it can be invoked by pressing a dedicated button in the Alexa application. On other devices where microphones are enabled and listening to surroundings, the device must be triggered with the wake word (most commonly "Alexa") Once the wake word detection occurs on the device, the device starts streaming all incoming voice signals to the cloud, where those signals are to be processed by Automatic Speech Recognition (ASR).

Automatic Speech Recognition (ASR): The main objective of Alexa ASR is to transform voice signals into text. Beyond this, it handles different challenges such as speed, volume, cadence, and clarity of speech (e.g. mumbling, accents, distortion of consonants) as well as background noises, slang words and archaisms. Combining three analysis models -- lexicon, acoustic, and language -- together helps ASR deal with the speech recognition challenges and allows it to decode the voice signal into the text with a high degree of precision. The ASR engine then sends the decoded words to the Natural Language Understanding (NLU) module.

Natural Language Understanding (NLU): The NLU module understands the users' intentions and the current context. It is typically a well-trained model that knows grammar rules of the language, is able to recognize the relationships between words, and extract the named entities. In practice, the NLU module is used on top of the skill's interaction model to match the user's speech with a corresponding intent, fill in the slot values, and pass the structured data to the fulfillment.

Interaction Model: The interaction model is the foundation of any voice application and is designed by the developers of an Alexa skill. It provides all the necessary information to understand and process the voice request. This information consists of invocation names, intents, sample utterances, and slots. After the intent is recognized and all required values are collected, the system can either respond back immediately or send the data together with the resolved slot values (i.e. variables) to the corresponding intent's handler to execute some additional logic.

Text-to-speech: Text-to-speech, also known as speech synthesis, is the ability of a machine to vocalize the written text. This process is opposite to the ASR.

Speech Synthesis Markup Language (SSML): SSML is an XML-based markup language recommended by the World Wide Web Consortium's (W3C) voice browser working group to provide guidance on how the machine should generate the speech. It allows controlling intonation, emphasis, rate of speech, and pronunciation.

Voice Recording and Privacy: As per the Amazon Alexa Terms of Use, it is possible that users' voice recordings may be stored for future use. While users are unable to prevent this storage process, they are able to permanently delete these recordings through their Amazon Alexa account. Amazon has provided instructions on how to do so here: https://www.amazon.com/gp/help/customer/display.html?nodeId=GHXNJNLTRWCTBBGW

Supplementary Note 3. Interaction Model

Melvin's Interaction Model describes the possible user intents and sample phrases used to trigger those intents. The Intent Handler is responsible for tracking state attributes based on the intent and slot values and then forming a structured query to retrieve analysis results from the Data Explorer Service. The final result is sent back to the user in the form of a speech response (in SSML) and visual content (in Alexa Presentation Language (APL)) which can be rendered in a supported Alexa device.

The Interaction Model is defined via a JSON file that models all intents (i.e., the actions through which users can interact with the skill). This also defines the parameters for each inquiry – including their types and the contextual phrases that Alexa needs for requesting them. The Interaction Model also contains many example sentences (utterances) for each intent that will be used by Alexa in recognizing user requests. The intents, interactions, and data types Melvin supports can be found in Supplementary Tables 1-3.

Supplementary Note 4. Intent Handler

This module handles requests that the user speaks as a command to an Alexa-enabled device. The Alexa Service then performs ASR and initial NLU and forwards for further processing (Supplementary Figure 1a-b). The requests received contain user session information, intent name, and slot values resolved by the Alexa Service. These slot values are often inaccurate due to a lack of domain-specific ASR and NLU capabilities in Alexa. Therefore,

Melvin attempts to map the slot value resolved by Alexa to its corresponding domain-specific attribute type and value via the OOVMS (Supplementary Figure 3a-c; see below for further details). Once the slot value is mapped, the Intent Handler will update its state by storing the corresponding attribute type and value (eg. `GENE` : `TP53`) as a session attribute using Alexa skills API. Storing state attributes as Alexa session attributes enables the Intent Handler to track the state of the dialogue flow since previously set session attributes are persisted across subsequent user requests in a multi-turn conversation. The Intent Handler will first validate the state change triggered by the user utterance before it attempts to formulate a structured query based on the current state (attribute values). For example, it is not possible to query for mutations (`DATA TYPE` : `MUTATIONS`) when both other attribute types (e.g. `GENE` and `CANCER TYPE`) are `NULL`. Once the state is validated, the Intent Handler will wrap the structured query in an HTTP request and send it over the internet to a remote endpoint where Data Explorer Service is hosted to retrieve the corresponding result. The result contains structured data which is used to generate a speech response based on a predefined template. In addition to the speech response, most replies contain a visual element that is delivered using Alexa APL technology. In addition, this module also supports sending the analyses generated during a session – as well as results from the last `N` (max 1000) interactions or within a specified timeframe (max `1` week) – back to the user via email. This feature utilizes the user's email id that is registered to the Alexa-enabled device.

Supplementary Note 5. Navigation State Tracking

The conversational dialogue flow for interrogating cancer genomics datasets is facilitated by implementing a finite state machine. This machine currently has three built-in attributes - `GENE`, `CANCER TYPE`, and `DATA TYPE` – that are tracked and updated during every user interaction. Notably, any number of attributes can be added to Melvin's finite state machine. For example, a `PROJECTS` attribute is already implemented, but – as proof-of-principle – only TCGA and BASIS are included within the current version of Melvin and TCGA interrogated by default. Users can update the attribute values by using sample phrases and arguments as defined in Melvin's Interaction Model. This approach allows users to take multiple navigation paths (i.e. conversations) to get the same result. This flexibility aims to increase overall usability and engagement.

Supplementary Note 6. Out-of-Vocabulary Mapper Service (OOVMS)

In order for voice user interfaces to be beneficial, they must be able to correctly hear and understand users. This is particularly challenging for Melvin as it primarily supports attributes (GENE, CANCER TYPE, and DATA TYPE) whose underlying values are often out-of-vocabulary (OOV). To overcome this challenge, we applied an ASR correction workflow which we refer to as the OOVMS. See Methods for details on OOVMS design, testing, and crowdsourced utterance collection. Below we provide additional details on the Pronunciation Quiz skill as well as the Custom Pronunciation Service.

Pronunciation Quiz Skill: We generated an orthogonal Alexa skill called Pronunciation Quiz (Supplementary Figure 2) to collect OOV term pronunciations as labeled data (i.e. "truth" data) that would be used to build the machine learning model to augment the OOVMS. This is a visual skill where the user would vocally repeat the term displayed on the screen. The only way to capture raw input in Alexa is through the `AMAZON.SearchQuery` slot type. By definition, an `AMAZON.SearchQuery` is a type of slot that is able to recognize less-predictable input that makes up the search query – similar to a standard search engine. A carrier phrase is required to

use this type of slot. In this case, the carrier phrase used was “that is.” Alexa recognizes all vocalization after a user says the carrier phrase as the OOV pronunciation and the ASR transcribed text is sent to the Pronunciation Quiz skill handler for further processing. Importantly, carrier phrases help ensure the integrity of utterances by reducing unintentional vocalizations.

Custom Pronunciation Web Portal: Users can create custom utterance mappings through the custom pronunciation web portal (Supplementary Figure 4). The web portal is integrated with Login-with-Amazon which allows third-party applications to access user profile details. The users are allowed to record their voice utterances for Melvin-supported attributes – including any CCDS gene. This can be performed via a personal computer, tablet, or mobile device with a microphone. Once the recording is uploaded, it is saved in an object store (i.e. AWS S3 Bucket). The metadata of the recording – e.g. the creation time, attribute type, attribute value, and its transcription via Amazon Poly – is stored in a separate utterances database. The records are tagged with the user’s email address retrieved from the Amazon user profile. When the custom utterances feature is enabled, Melvin will first search user-specific utterances for any matching records to resolve the query utterance before calling the OOVMS. These lookups are user-specific since Alexa’s account linking feature allows Melvin to retrieve the user’s email address from the associated Amazon profile. Overall, this service – along with the OOVMS – vastly improves Melvin’s ability to correctly understand when users vocalize supported cancer genomics terms.

Supplementary Note 7. Data Explorer Service

The Data Explorer Service is an umbrella term referring to the computational framework and modules that do most of the heavy lifting in terms of Melvin’s data analytics. It is developed using microservices architecture, which makes it scalable, interoperable, and loosely coupled. It is composed of multiple subcomponents - Melvin data model, analytics modules, plotting modules, and Melvin REST API. It provides standalone API endpoints for statistical and visual responses to incoming query packets. The data warehousing mainly comprises raw datasets cleaned and processed into Amazon Aurora (RDS) which is an AWS-native relational database service.

Various functions are present within the service to generate answers to analytical/computational queries pertinent Melvin’s supported attribute types and values. Every analysis subsystem has its own visualization function that produces relevant plots to accompany Melvin’s speech responses. A data access object submodule queries the RDS and pipes the data through a plotting module and a computational module. This is implemented as a simultaneous, two-pronged execution. While Alexa’s internal timeout is set at 8 seconds for response generation, the computation and plotting are completed at an average of 3 seconds with the plots taking a few additional seconds to render as the speech response is enunciated. With state-based navigation and incremental dialogue flow, the Data Explorer Service enables robust addition of attribute types and switching between attribute values. This scalable and efficient analytical infrastructure results in data-driven insights through continuous, real-time interactions.

Database design: The data within Melvin’s Data Explorer Service (see Methods) is hosted as a MySQL database in the Aurora RDS. The features necessary for Melvin’s data model are extracted, transformed, and loaded (ETL) as tables in the Aurora RDS. The analytics modules use a Python-MySQL Object Relational Mapper toolkit called SQLAlchemy to build SQL queries to access data. This mode of interaction makes data ingestion/modification and maintenance of Melvin’s data model scalable and extensible. All main tables are indexed for patient, gene, and cancer type fields for faster querying and retrieval. Pre-computed data tables were generated

for a small subset of Melvin states (e.g. gene expression summaries across all cancer types) to ensure that Melvin can receive requests and relay responses in under 8 seconds.

Supplementary Note 8. End-to-end testing framework

The robustness and veracity of Melvin's back-end – including a detailed assessment of utterance mapping accuracy – is ensured through a comprehensive end-to-end testing framework built by leveraging test automation tools offered by Bespoken. The test cases were created by generating synthetic audio clips using Amazon Polly to simulate real interactions with Melvin. The Bespoken testing tool enables test cases to be run concurrently using virtual devices in the cloud, thus reducing the time to complete a full regression test cycle to less than 15 minutes. These tests were integrated into a continuous integration and deployment pipeline setup on GitLab. As the ASR underlying Amazon Alexa may be updated over time, this framework allows us to continuously and frequently assess the performance of the OOVMS and tune it when necessary.

Supplementary Note 9. Enhancing User Experience

As previously discussed, Melvin's novel design has major components that substantially improve user experience (e.g. state-based conversations, multimodal responses, OOVMS, Custom Pronunciation Web Portal, and exporting results via email). Melvin also implements numerous minor features to further enhance its utility and reduce friction. We have customized Alexa reprompts and APL parameters to keep Melvin sessions open for an extended amount of time. This allows users to sufficiently digest results and formulate their next query. This is exceptionally useful in the conference room setting such as laboratory meetings or molecular tumor boards (Supplementary Figure 5). If an Alexa-enabled device is connected to a display (e.g. projector, LED television, etc.), Melvin's visual output will persist until someone engages Melvin with a new request or closes the session. This enables Melvin to serve as an augmented intelligence tool to help teams interpret cancer genomics findings. For computationally intensive queries that take longer than usual, Melvin will generate progressive responses to return an intermediate response of "I'm still working on that. Please wait." This allows us to let users know to expect their results momentarily. Melvin keeps track of each interaction in the current session and allows users to navigate back to the previous state by using the phrase "go back". Users are also able to restore their most recent session and resume their analysis from where they left off by using any device that is connected to their Alexa account. At any point, a user is able to clean the state and history of the current session by using the phrase "start over". Lastly, by enabling brief mode, users can limit Melvin's vocal responses to critical information only – streamlining interactions and further minimizing time to result.

Supplementary Tables

Supplementary Table 1 | Supported intents within Melvin. The intent names – as well sample invocation phrases – are provided.

**Intent Name**	**Sample Phrases**
NavigateJoinFilterIntent	"how about {query}",<br>"what about {query}",<br>"show me in {query}",<br>"show me for {query}",<br>"show me {query}",<br>"tell me about {query}",<br>"tell me {query}",<br>"look at {query}",<br>"look up {query}"
NavigateEmailIntent	email that to me<br>send that to me<br>email last {count} results<br>send last {count} results<br>email results within {duration}<br>send results within {duration}

Supplementary Table 2 | Supported data types within Melvin. These can be accessed by uttering the data type name explicitly or any of the provided alternative names.

**Data type Name**	**Alternatives**
Mutations (SNVs & indels)	
Indels	
SNVs	Single nucleotide variants
Domains	● Protein domains<br>● Most affected domains
CNAs	● Copy numbers<br>● Copy number alterations<br>● Copy number variations<br>● Copy number changes
Gains	● Amplifications<br>● Copy number gains<br>● Copy number amplifications
Losses	● Deletions<br>● Copy number losses<br>● Copy number deletions
Gene Expression	● Expression<br>● RNA expression<br>● mRNA expression

Supplementary Table 3 | Supported interactions within Melvin. The trigger phrases for each interaction – as well their alternatives – are provided.

Trigger Phrase	Alternatives	Purpose
open *Melvin Genomics*		This is to invoke the skill in Alexa after it has been enabled.
tell me about	● show me<br>● how about<br>● what about<br>● show me in<br>● show me for<br>● look at<br>● tell me	This is to start exploring or switching between different genes, cancer types or data types.
gene definition	● what does it do?<br>● what does this gene do?<br>● what does this protein do?<br>● definition<br>● protein definition<br>● what is its function?	This is to get axillary information about a gene, including its known function, from NCBI.
drug options	● is it targetable?<br>● is it druggable?<br>● is it actionable?<br>● is this gene targetable?<br>● is this gene druggable?<br>● is this gene actionable?<br>● is this protein targetable?<br>● is this protein druggable?<br>● is this protein actionable?	This is to know if the gene is druggable based on USFDA reporting.
compare	● compare with	This is to compare between two genes, two data types, or two cancer types.
split-by	● split by	Within a cancer type, this is to assess how the expression of one gene varies in relation to mutations or copy number changes (in the same gene or another gene).
email that to me	● send that to me<br>● email that to me<br>● email last *n* results (max 1000)<br>● send last *n* results (max 1000)<br>● email results within *time* (max 1 week)<br>● send results within *time* (max 1 week)	This is to get the last analysis sent to your email.
enable/disable custom	● turn on custom mappings	This is to enable/disable custom

mappings	• enable custom mappings<br>• turn off custom mappings<br>• disable custom mappings	whitelisting of your own pronunciations of genes, cancer types, or data types via the Custom Pronunciation Web Portal.
reset	• start over<br>• clear everything<br>• clear all	This is to reset the state and start from scratch.
step back		This is to go back one step in the conversation.
resume session	restore session	This is to restore the previous session where the user left off.
repeat that	• show last analysis<br>• where am I?<br>• repeat last	This is to reiterate the last result and know the current user state in the conversation.
stop	• goodbye<br>• cancel	This exits the skill.
help	• help me<br>• can you help me?	This is to know more about how to use the skill.
brief mode (short responses)	• turn on brief mode<br>• enable brief mode<br>• turn off brief mode<br>• disable brief mode	Indicates to Melvin that shorter audio responses are preferred whenever possible (i.e. generates a shorter version of voice results).

Supplementary Table 4 | Examples of possible *compare* and *split-by* analyses. Melvin supports special types of analyses that allow users to compare the current state to another or partition the current state based on categorical attributes (i.e. *split-by*).

Navigation State Attributes	Operation (Intent)	Auxiliary Attributes
1. `GENE`: *PIK3CA*<br>2. `CANCER TYPE`: breast cancer<br>3. `DATA TYPE`: copy number alterations	*Compare*	1. `DATA TYPE`: mutations
1. `GENE`: *PIK3CA*<br>2. `CANCER TYPE`: breast cancer<br>3. `DATA TYPE`: copy number alterations	*Compare*	1. `GENE`: *BRCA1*
1. `GENE`: *PIK3CA*<br>2. `CANCER TYPE`: breast cancer<br>3. `DATA TYPE`: expression	*Compare*	1. `CANCER TYPE`: ovarian cancer
1. `GENE`: *PIK3CA*<br>2. `CANCER TYPE`: breast cancer	*Compare*	1. `CANCER TYPE`: ovarian cancer
1. `GENE`: *PIK3CA*<br>2. `CANCER TYPE`: breast cancer<br>3. `DATA TYPE`: expression	*Split-by*	1. `DATA TYPE`: mutations<br>2. `GENE`: *TP53*

Supplementary Figures

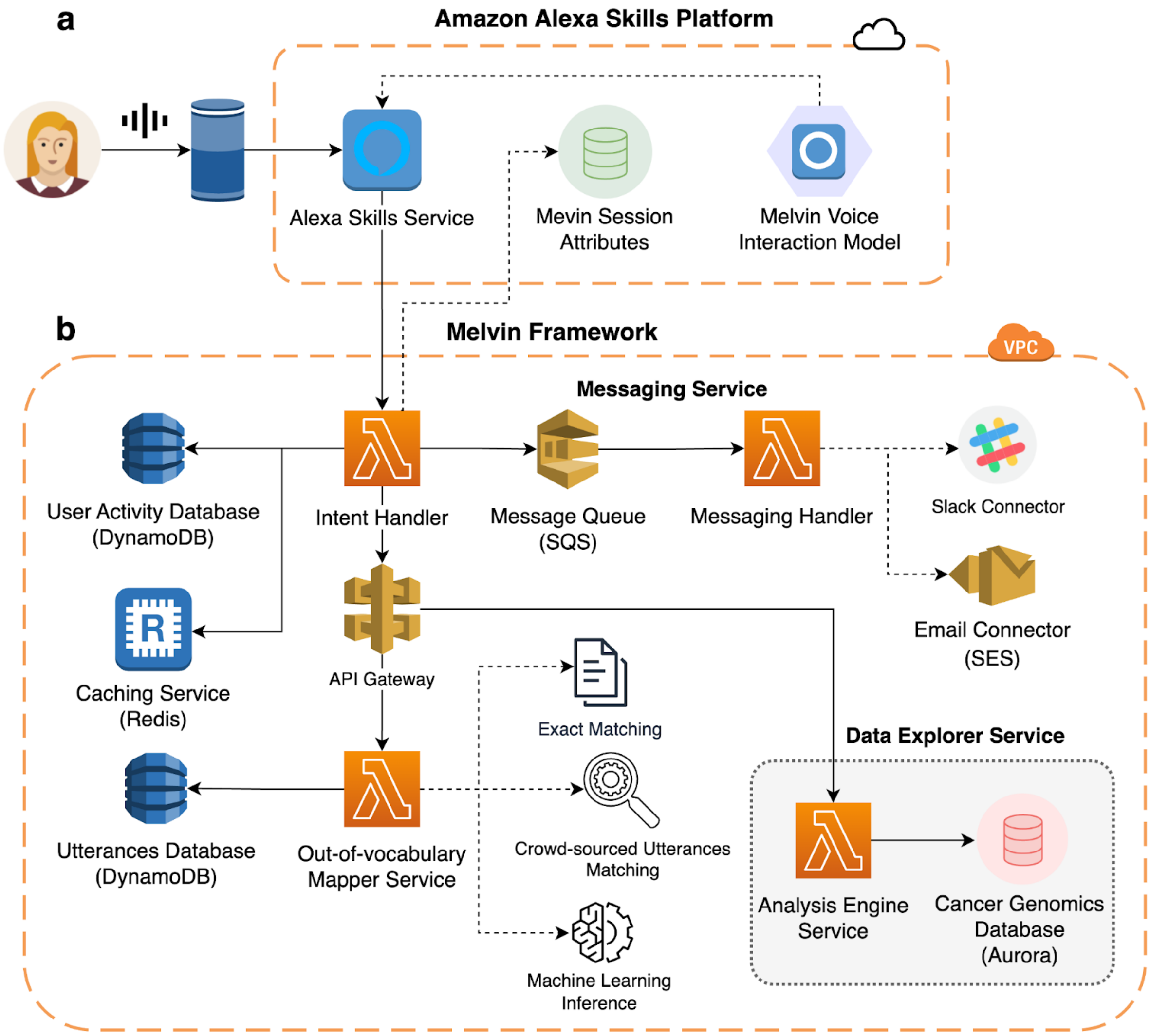

Supplementary Figure 1 | Cloud-based system design of Melvin. (a) Depicts the capabilities provided by Amazon cloud. Speech-to-text conversion of user utterances is performed using built-in ASR capabilities in the Alexa cloud platform. This is followed by intent identification and extraction of slot values as defined in the Melvin voice interaction model. **(b)** Depicts services developed and deployed on AWS that are consumed by the Melvin skill. Both Analysis Engine Service and Out-of-vocabulary Mapper Service components expose RESTful APIs through API Gateway and are implemented as serverless functions. The cancer genomics database is provisioned using Amazon Aurora database engine which is highly robust and dynamically scales according to the workload. Databases containing user activity and crowdsourced utterances are provisioned using Amazon DynamoDB cloud-based NoSQL database platform that provides high-throughput and low-latency operations.

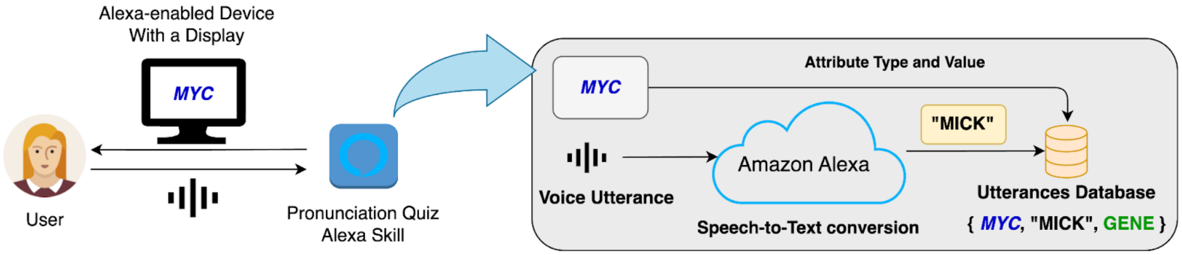

Supplementary Figure 2 | Crowdsourcing utterances through the Pronunciation Quiz Alexa skill. Users were shown a flash card containing an attribute value for a `GENE`, `CANCER TYPE`, or `DATA TYPE` and asked to pronounce them. The captured voice utterance (pronunciation) was converted to text via Alexa platform's speech-to-text capabilities and received by the Pronunciation Quiz Alexa skill. These speech-to-text representations of the pronunciations (e.g. "MICK") were recorded in a database with the attribute type (e.g. `GENE`) and attribute value (e.g. "*MYC*").

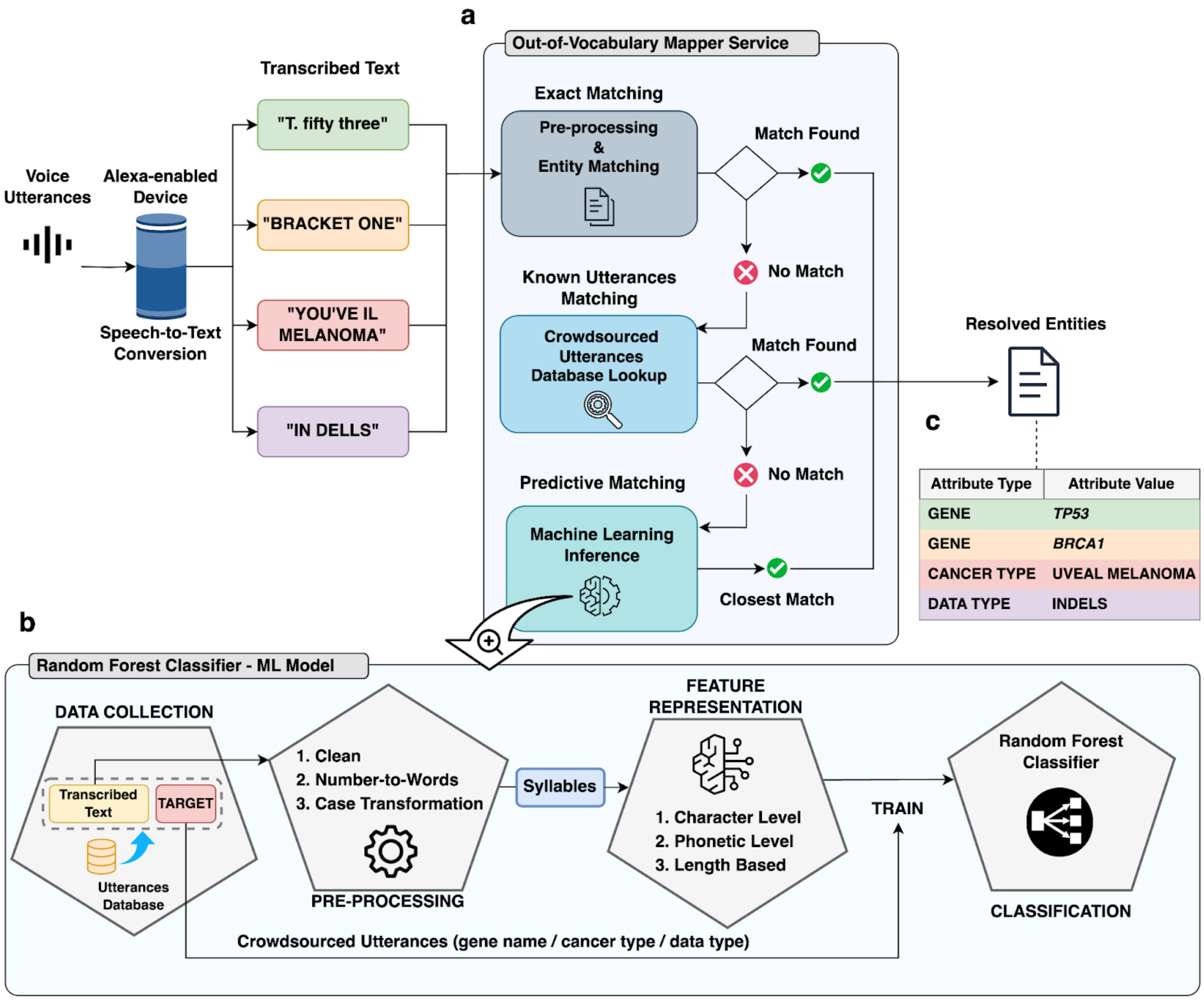

Supplementary Figure 3 | Utterance processing workflow for the Out-of-vocabulary Mapper Service (OOVMS). (a) Transcribed user utterances are first subject to exact matching in which the incoming query text is cleaned, case transformed, lemmatized, and cross-referenced to a predefined set of valid attributes. If an exact match is not found, then a database lookup is performed to find a matching labeled utterance that has been previously captured and associated with a valid attribute (i.e. crowdsourced utterance). When no crowdsourced utterance is found, a machine learning model (i.e. random forest classifier) is used to best classify the input query. **(b)** Depicts the construction of the random forest classifier used by the OOVMS. During training data collection, Pronunciation Quiz was leveraged to crowdsource utterances to be used as labeled data. These crowdsourced utterances were pre-processed by applying a series of lexical transformations and represented as flat features. A random forest classifier was trained to learn the mapping between the utterance transcriptions and target attribute values. This model classifies unobserved utterances (i.e. those absent from the crowdsourced utterances database) to the closest matching valid attribute **(c).** The final, resolved output is an attribute value and its associated attribute type.

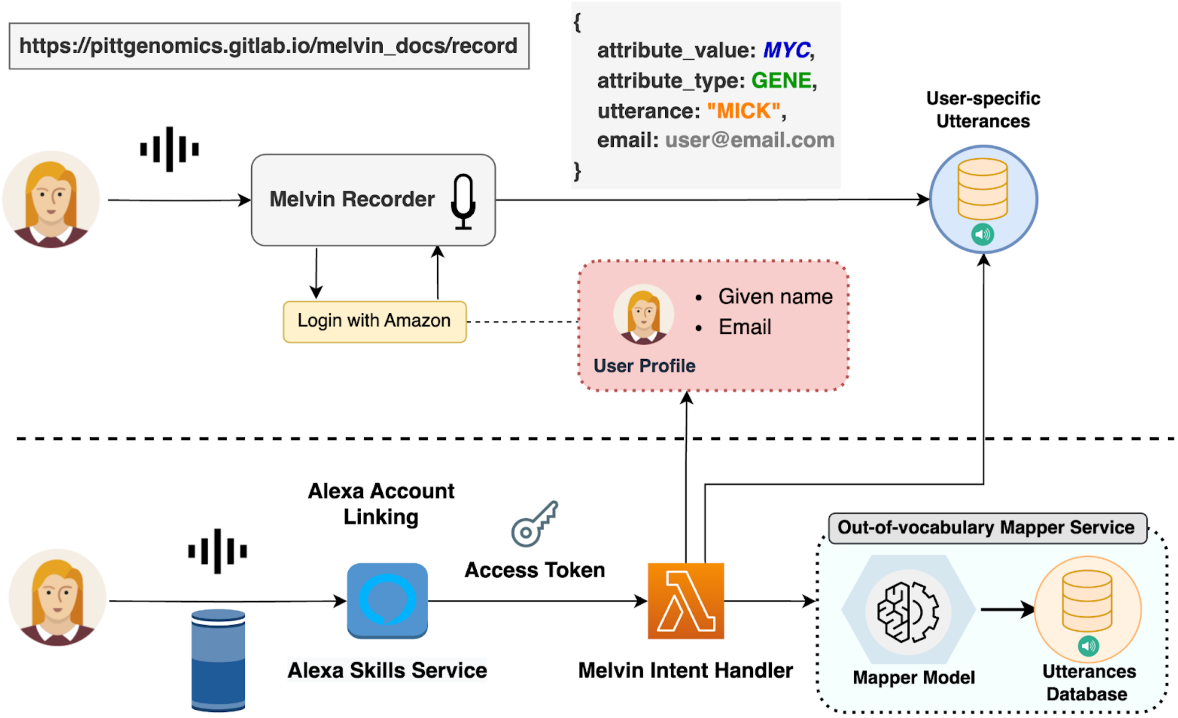

Supplementary Figure 4 | Creating user-specific utterance mappings through Custom Pronunciation Web Portal. Through a web-based interface, users can access the Melvin Recorder Service. Here they can provide personalized pronunciation for any attribute value (i.e. a `GENE`, `CANCER TYPE`, or `DATA TYPE`) of interest. This includes all CCDS genes. These utterances are stored in an AWS DynamoDB database and connected to user accounts through Login with Amazon and Alexa Account Linking. When users have the custom mappings feature enabled, Melvin will first determine if the ASR transcribed text matches any user-specific pronunciations generated via the Custom Pronunciation Web Portal. If there is no match, the transcribed text will be mapped to a `GENE`, `CANCER TYPE`, or `DATA TYPE` via the Out-of-vocabulary Mapper Service (OOVMS).

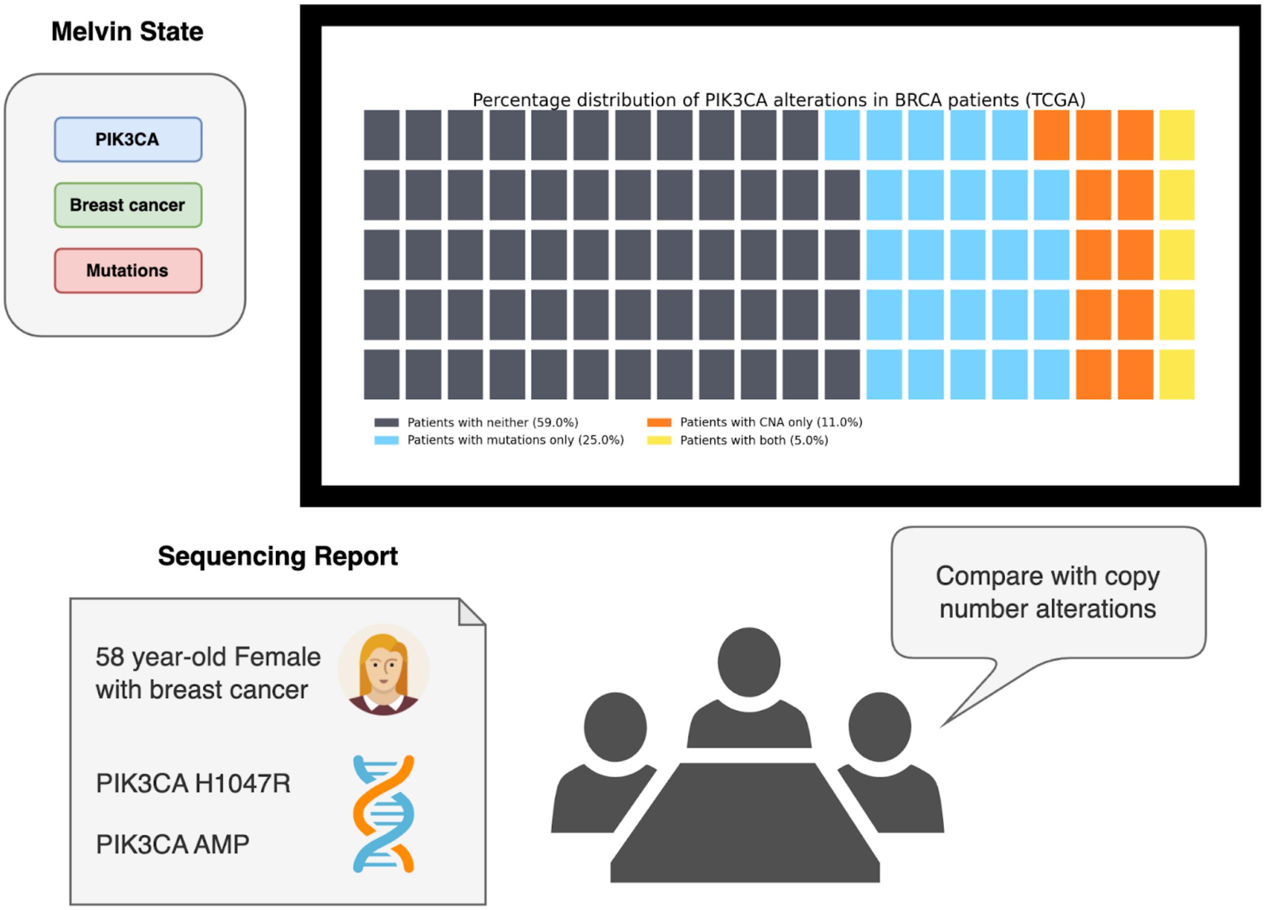

Supplementary Figure 5 | Melvin deployed in a laboratory meeting or molecular tumor board setting. By connecting a conference room projector and sound system to an Alexa-supported device, Melvin's results are projected to the audience. Additionally, multiple individuals within the venue may interact with Melvin via voice – even within the same conversation.
