# Supplementary material for: Melvin is a conversational voice interface for cancer genomics data
Source: Commun Biol. 2024 Jan 5;7:30. doi: 10.1038/s42003-023-05688-z (PMC10770357; doi:10.1038/s42003-023-05688-z)
Supplement: Supplementary file 3 — Description of Additional Supplementary Files [file 42003_2023_5688_MOESM3_ESM.pdf]

## Description of Additional Supplementary Files

**File name:** Supplementary Data 1
**Description:** Cancer-associated genes (n = 723) that were collected from the Cancer Gene Census.

**File name:** Supplementary Data 2
**Description:** List of GENE, CANCER TYPE, and DATA TYPE attribute values (and associated synonyms) whose pronunciations were crowdsourced via Pronunciation Quiz.

**File name:** Supplementary Movie 1
**Description:** Obtaining the TP53 mutation rate in TCGA breast cancer (Figure 1 demo).

**File name:** Supplementary Movie 2
**Description:** Exploring TP53 and PIK3CA alterations in BASIS.

**File name:** Supplementary Movie 3
**Description:** Definition and actionability of the RET gene.

**File name:** Supplementary Movie 4
**Description:** Multi-turn conversations via state-based dialogue flow (Figure 2a demo).

**File name:** Supplementary Movie 5
**Description:** A compare and split-by interaction (Figure 2b-c demo).
