# Supplementary material for: Melvin is a conversational voice interface for cancer genomics data
Source: Commun Biol. 2024 Jan 5;7:30. doi: 10.1038/s42003-023-05688-z (PMC10770357; doi:10.1038/s42003-023-05688-z)
Supplement: Supplementary file 11 — Reporting Summary [file 42003_2023_5688_MOESM11_ESM.pdf]

nature portfolio

Corresponding author(s): Jason J Pitt

Last updated by author(s): Nov 18, 2023

Reporting Summary

Nature Portfolio wishes to improve the reproducibility of the work that we publish. This form provides structure for consistency and transparency in reporting. For further information on Nature Portfolio policies, see our Editorial Policies and the Editorial Policy Checklist.

Statistics

For all statistical analyses, confirm that the following items are present in the figure legend, table legend, main text, or Methods section.

n/a	Confirmed	
☒	☐	The exact sample size (*n*) for each experimental group/condition, given as a discrete number and unit of measurement
☒	☐	A statement on whether measurements were taken from distinct samples or whether the same sample was measured repeatedly
☒	☐	The statistical test(s) used AND whether they are one- or two-sided<br>*Only common tests should be described solely by name; describe more complex techniques in the Methods section.*
☒	☐	A description of all covariates tested
☒	☐	A description of any assumptions or corrections, such as tests of normality and adjustment for multiple comparisons
☒	☐	A full description of the statistical parameters including central tendency (e.g. means) or other basic estimates (e.g. regression coefficient) AND variation (e.g. standard deviation) or associated estimates of uncertainty (e.g. confidence intervals)
☒	☐	For null hypothesis testing, the test statistic (e.g. *F*, *t*, *r*) with confidence intervals, effect sizes, degrees of freedom and *P* value noted<br>*Give P values as exact values whenever suitable.*
☒	☐	For Bayesian analysis, information on the choice of priors and Markov chain Monte Carlo settings
☒	☐	For hierarchical and complex designs, identification of the appropriate level for tests and full reporting of outcomes
☒	☐	Estimates of effect sizes (e.g. Cohen's *d*, Pearson's *r*), indicating how they were calculated

Our web collection on statistics for biologists contains articles on many of the points above.

Software and code

Policy information about availability of computer code

Data collection	No custom code was used for genomic data collection. Details on any genomic data formatting after downloading from public sources is explained within the Methods or Supplementary Information. The codebases underlying the Pronunciation Quiz skill and intent handler, which were used to gather crowdsourced utterances for optimizing the Out-of-Vocabulary Mapper Service (OOVMS), have been made publicly available on GitHub and Zenodo. Links to the repositories are provided in the Code Availability statement.
Data analysis	The code underlying genomic analyses that Melvin can perform has been provided as a public GitHub repository (https://github.com/pittlab-genomics/Melvin_Alexa_Intent_Handler). The Python libraries and R packages, along with their versions, are provided in the Statistics and Reproducibility subsection of the Methods.

For manuscripts utilizing custom algorithms or software that are central to the research but not yet described in published literature, software must be made available to editors and reviewers. We strongly encourage code deposition in a community repository (e.g. GitHub). See the Nature Portfolio guidelines for submitting code & software for further information.

Data

Policy information about availability of data

All manuscripts must include a data availability statement. This statement should provide the following information, where applicable:

- Accession codes, unique identifiers, or web links for publicly available datasets
- A description of any restrictions on data availability
- For clinical datasets or third party data, please ensure that the statement adheres to our policy

Data Availability statement: "All data within Melvin's Explorer Service was taken from publicly available sources. Details of these sources as well as dataset release versions can be found in the Methods section."

Human research participants

Policy information about studies involving human research participants and Sex and Gender in Research.

Reporting on sex and gender	We did not perform any reporting based on sex and gender.
Population characteristics	As all participants are from The Cancer Genome Atlas (TCGA) and Breast Cancer Somatic genetics Study (BASIS), all genomic data within Melvin's Explorer Service represents malignant tissue from individuals with a cancer diagnosis.
Recruitment	No participant recruitment was performed within this study.
Ethics oversight	All genomic data sources are publicly available and all ethical considerations are under the purview of those data providers. No ethics approvals were required from the institutions affiliated with this study's authors.

Note that full information on the approval of the study protocol must also be provided in the manuscript.

Field-specific reporting

Please select the one below that is the best fit for your research. If you are not sure, read the appropriate sections before making your selection.

☒ Life sciences ☐ Behavioural & social sciences ☐ Ecological, evolutionary & environmental sciences

For a reference copy of the document with all sections, see nature.com/documents/nr-reporting-summary-flat.pdf

Life sciences study design

All studies must disclose on these points even when the disclosure is negative.

Sample size	The total number of samples included in the Melvin Explorer Service were 11,271 (with at least one DATA TYPE) and 344 from TCGA and BASIS, respectively.
Data exclusions	For TCGA, a participant needed to have either mutational, copy number alteration, or expression data to be included within the Melvin Data Explorer Service. For a BASIS participant to be included in the Melvin Explorer Service, we required each to have both mutational and copy number alteration data.
Replication	Through end-to-end software testing, we have ensured our computational framework provides reproducible results over genomic data. However, no replication was required as our study does not report novel genomic findings requiring hypothesis testing.
Randomization	Within the Melvin Explorer Service, groups were defined by preexisting labels provided by the data sources (e.g. dataset, cancer type, etc.). No randomization was necessary or performed by the study authors.
Blinding	The development of Melvin did not require hypothesis testing and consequently did not require any blinding of researchers.

Reporting for specific materials, systems and methods

We require information from authors about some types of materials, experimental systems and methods used in many studies. Here, indicate whether each material, system or method listed is relevant to your study. If you are not sure if a list item applies to your research, read the appropriate section before selecting a response.

Materials & experimental systems

n/a	Involved in the study
☒	☐ Antibodies
☒	☐ Eukaryotic cell lines
☒	☐ Palaeontology and archaeology
☒	☐ Animals and other organisms
☒	☐ Clinical data
☒	☐ Dual use research of concern

Methods

n/a	Involved in the study
☒	☐ ChIP-seq
☒	☐ Flow cytometry
☒	☐ MRI-based neuroimaging
